# Polysiloxane ionic liquids as good solvents for β-cyclodextrin-polydimethylsiloxane polyrotaxane structures

**DOI:** 10.3762/bjoc.8.184

**Published:** 2012-09-24

**Authors:** Narcisa Marangoci, Rodinel Ardeleanu, Laura Ursu, Constanta Ibanescu, Maricel Danu, Mariana Pinteala, Bogdan C Simionescu

**Affiliations:** 1 Centre of Advanced Research in Bionanoconjugates and Biopolymers, “Petru Poni” Institute of Macromolecular Chemistry, 700487 Iasi, Romania; 2 Department of Natural and Synthetic Polymers, “Gheorghe Asachi” Technical University of Iasi, 700050 Iasi, Romania

**Keywords:** cyclodextrins, imidazolium salt, ionic liquid, polyrotaxanes, polysiloxanes

## Abstract

An ionic liquid based on polydimethylsiloxane with imidazolium salt brushes was synthesized as a good solvent for β-cyclodextrin-polydimethylsiloxane rotaxane. As expected the PDMS-Im/Br ionic liquid had a liquid-like non-Newtonian behavior with rheological parameters dependent on frequency and temperature. The addition of rotaxane to the ionic liquid strengthened the non-Newtonian character of the sample and a type of stable liquid-like network was formed due to the contribution of weak ionic interactions. The structure is stable in the 20 to 80 °C domain as proved by the oscillatory and rotational rheological tests.

## Introduction

Ionic liquids (ILs) are environmentally friendly solvents with great potential for chemical and nonchemical applications due to their low melting points, nonvolatile and noncorrosive properties at room temperature. They possess good conductivities [1–2] and often they present a good thermal stability up to 400 °C [1,3]. The potential applications of ILs made them the subject of a number of works that showed their use as solvents or as solvents for synthesis and catalysis [4–6]. In this context ILs, which have polar and nonpolar regions, could play an important role in the field of supramolecular organization of different supramolecular structures (such as polyrotaxanes or supermolecules formed by ILs with different host molecules), leading to interesting phenomena, properties and applications [7–12]. This includes the dissolution of cellulose and cyclodextrins (CDs) with ILs [12], synthesis of ILs containing slide-ring gels [7], synthesis of IL-CD inclusion complexes [10], etc.

Polyrotaxane structures based on cyclodextrins and different linear (co)polymers are well known as supramolecular ensembles. They consist of cyclodextrin molecules whose hydrophobic cavities are penetrated by a linear polymer chain terminating with bulky stoppers, which prevent the macrocycle from slipping out [13–22]. Unfortunately, the properties and mechanisms of CD-polymer polyrotaxanes have been rarely evaluated due to a lack of good solvents. In general, the CD-polymer polyrotaxanes are soluble only in DMSO and aqueous sodium hydroxide solution, even if each component of their structure is soluble in a large number of solvents [8]. Research for new solvents has led to the discovery of ionic liquids as a good option for pseudo- or polyrotaxane structures [7–8,17–18]. It should be also mentioned that the mobility of the carrier ions of ILs decreases when their glass-transition temperature (*T*_g_) increases [23]. In this respect, we have synthesized ILs based on polydimethylsiloxane with imidazolium salt brushes (PDMS-Im/S) with low *T*_g_ values in order to avoid a macroscopic phase separation in a mixture of ILs and CD-polydimethylsiloxane polyrotaxane structures. Also, in the present study, we report how the rheological properties are influenced when CD-polydimethylsiloxane rotaxane is dissolved in PDMS-Im/S ionic liquid.

## Results and Discussion

**Scheme 1:**
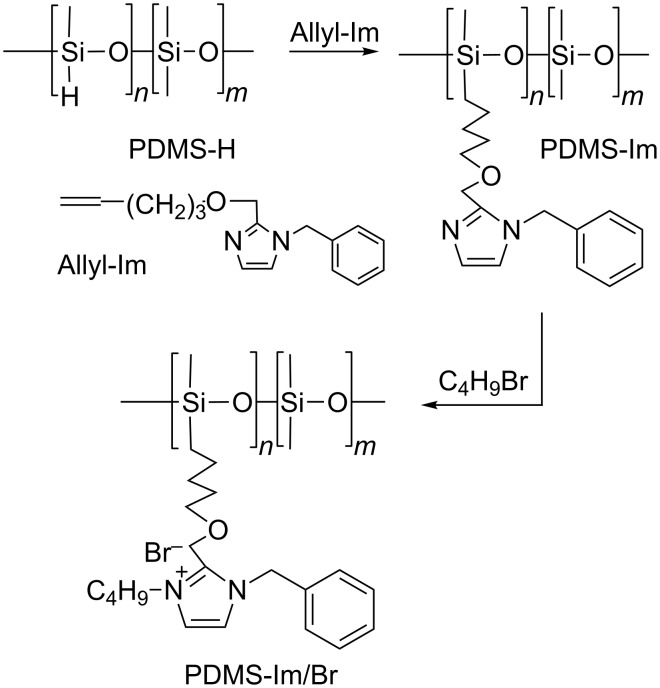
Synthesis of PDMS-Im/Br ionic liquid.

A class of polymers that possesses a unique combination of properties, such as very low *T*_g_ values, high chain flexibility, good thermal, oxidative and UV stability, high gas permeability, surface activity, hydrophobicity, etc., is the class of polysiloxanes, especially polydimethylsiloxanes [24]. It is worth mentioning that ionic liquids based on polydimethylsiloxane and imidazolium salt groups have been used for capillary gas chromatography [25–26] and as biocides [27]. Furthermore, one of our interests is to combine the properties of polydimethylsiloxanes with the versatility of synthetic approaches in designing well-defined macromolecular systems [17,21,27]. In this context, we synthesized polysiloxanes with a pendant imidazolium bromide derivative (PDMS-Im/Br) through a multistep procedure (Scheme 1) as an ionic liquid, which serves as a good solvent for β-CD-polydimethylsiloxane polyrotaxane (PRot).

The copolymer PDMS-Im is characterized by a (CH_3_)_2_SiO/Im(CH_3_)SiO molar ratio of 3/1, as determined from its ^1^H NMR spectrum. PRot is characterized by a *M*_n_ = 1250 of polydimethylsiloxane (PDMS) and β-CD/PDMS chain ratio of 2/1, both determined from the integrals of characteristic peaks in its ^1^H NMR spectrum [21].

**Figure 1:**
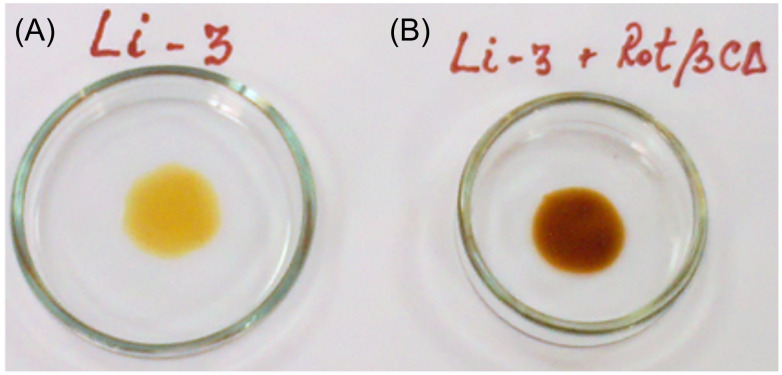
Appearance of (A) pure PDMS-Im/Br ionic liquid; (B) PDMS-Im/Br ionic liquid containing 1 wt % PRot.

After 24 hours of stirring at 90 °C under nitrogen atmosphere, the mixture of 10 wt % PRot with PDMS-Im/Br turned into a viscous clear solution, suggesting a complete dissolution. Cooling the sample to room temperature in a dry box caused an increase in the apparent viscosity; it should also be mentioned that the mixture remained clear for the next five weeks (Figure 1).

**Figure 2:**
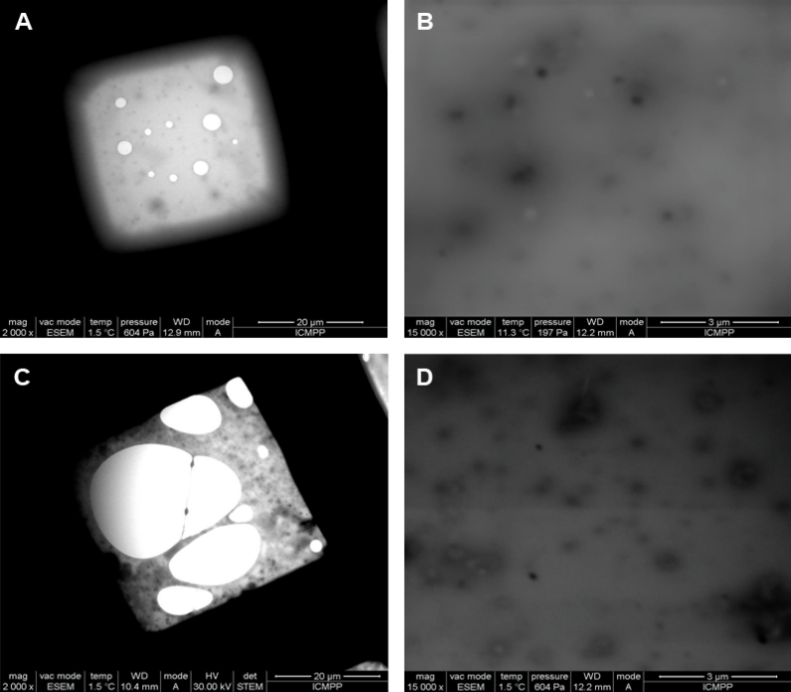
Wet-STEM images at 30 kV in bright field mode of: PDMS-Im/Br ionic liquid (A,B) and mixture of PDMS-Im/Br with PRot (C,D).

This observation indicates the complete dissolution of PRot in PDMS-Im/Br ionic liquid. This may be due to the disruption of the intermolecular hydrogen bonds that exist in β-CD-polymer polyrotaxane structures by the ionic liquid [9]. In addition, an ordered morphology was observed from wet-STEM images of the mixture, which also indicates a good dissolution of PRot in the PDMS-Im/Br ionic liquid (Figure 2).

**Figure 3:**
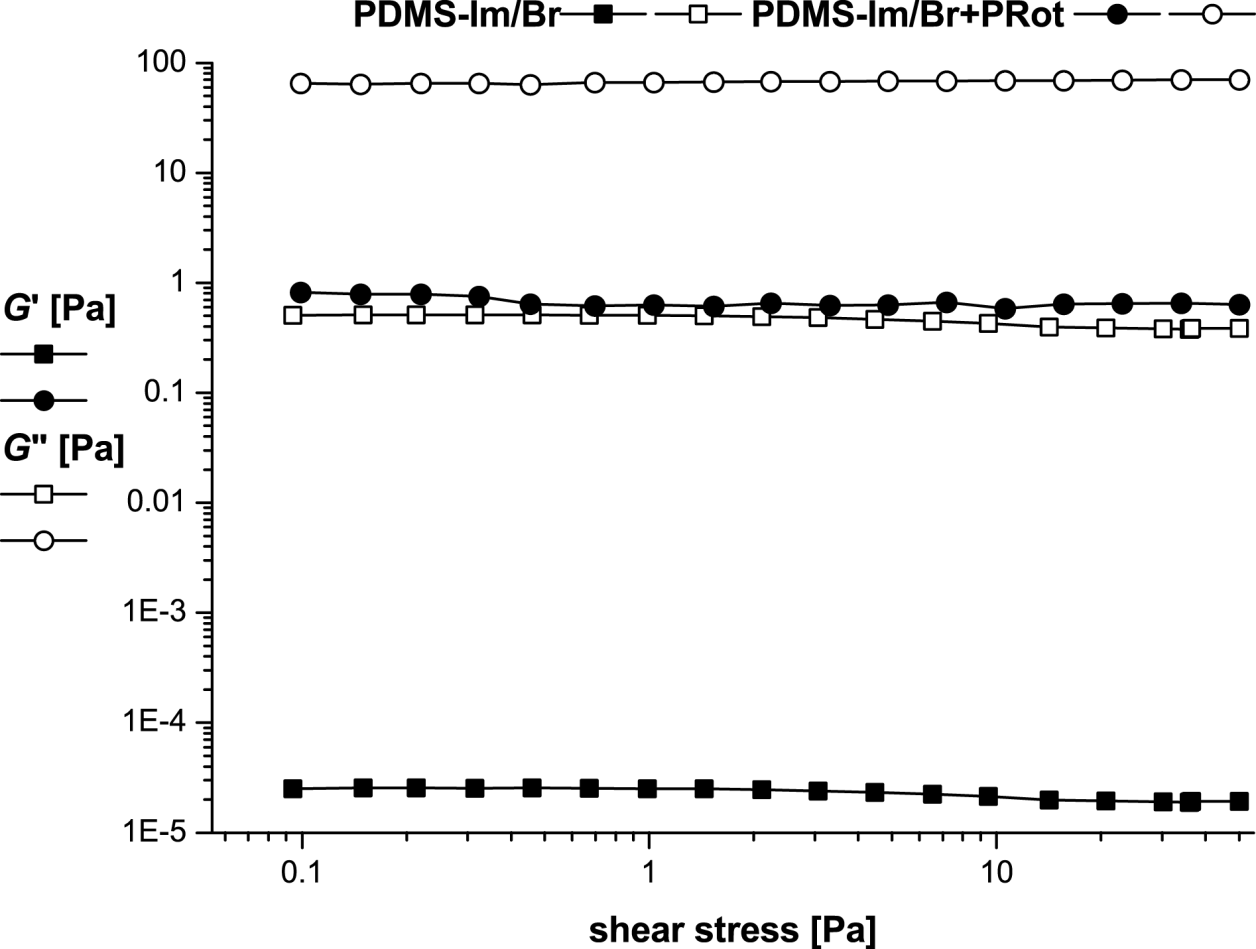
Amplitude sweep results for PDMS-Im/Br and PDMS-Im/Br+PRot at 25 °C.

Apparently, the size of the morphology is maintained in PDMS-Im/Br with PRot mixture (Figure 2C,D) when we compare it with those corresponding to the ionic liquid (Figure 2A,B). The rheological properties of the PDMS-Im/Br ionic liquid and its mixture with PRot give important information on the interaction between IL and PRot and can explain the dissolution behavior of polyrotaxane in the ionic liquid [28–33]. The rheology of the mixture was studied by using both oscillatory and rotational shear measurements. A solution with a concentration of 10 wt % was used for all tests. The first test, prior to all the oscillatory measurements, was the strain (amplitude) sweep at a fixed frequency of 1 Hz and a shear stress varying from 0.1 to 50 Pa. In Figure 3, the storage modulus *G*’, the loss modulus *G*”, and the phase shift angle δ, as a function of shear stress, are presented.

As long as *G*” > *G*’ for the entire experimental domain, we can suppose a stable liquid-like structure with an extended linear viscoelastic plateau. The limits of the linear viscoelastic region (LVR) were established at 7.5 Pa for the ionic liquid and 43.7 Pa for the ionic liquid with rotaxane. The presence of rotaxane, therefore, extended the LVR domain. Even if, as usual, the amplitude sweep is used only to determine the limiting values of strain or shear stress, which are necessary for all the subsequent oscillatory tests, interesting data regarding the rheological behavior of solutions of complex fluids can be also obtained.

**Figure 4:**
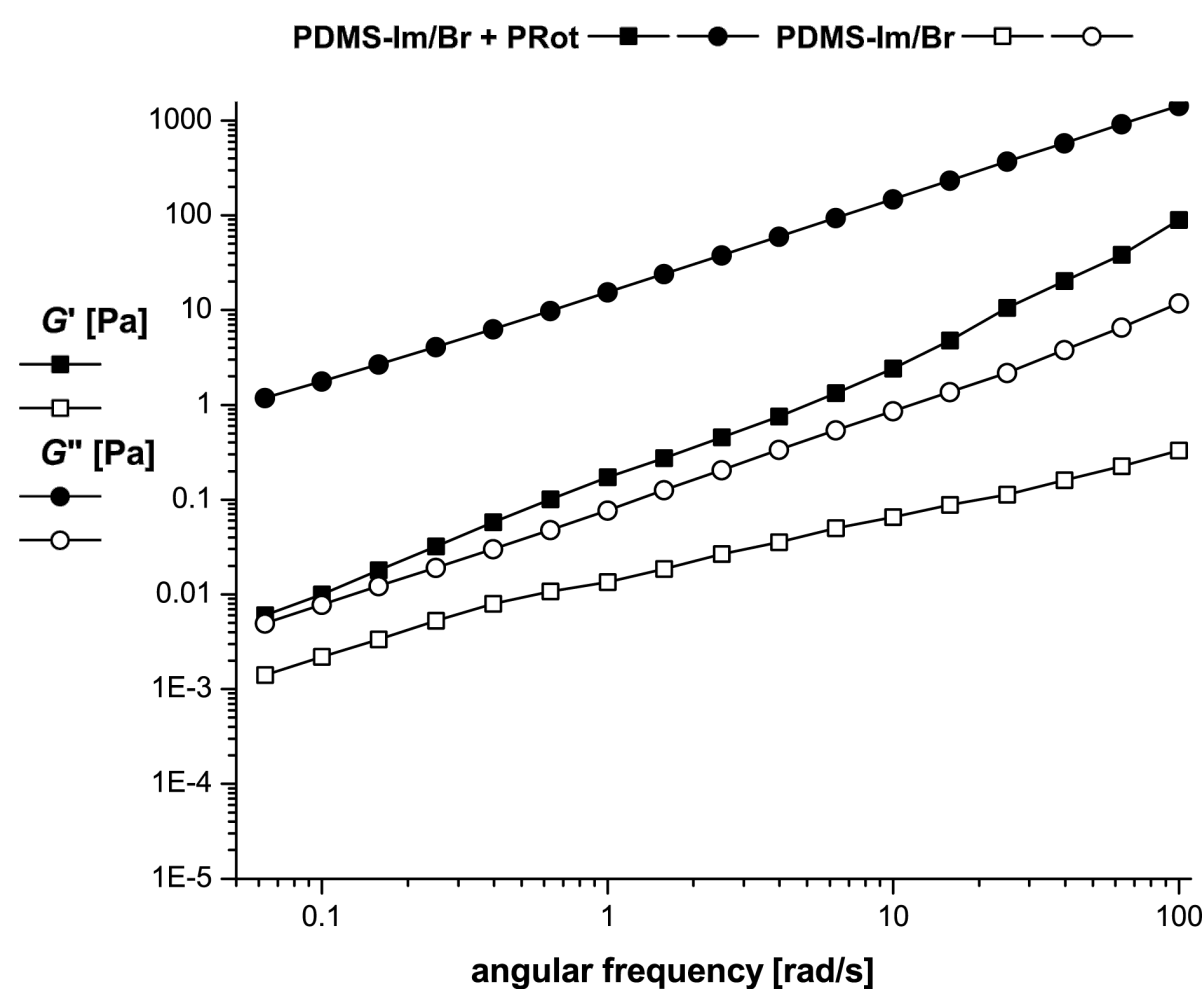
Storage (*G*’) and loss (*G*”) moduli dependence on frequency for PDMS-Im/Br and PDMS-Im/Br+PRot at 25 °C.

As mentioned in the literature [28], different strain-dependent behaviors may be recognized in the amplitude sweeps for various types of polymer solutions taking into consideration their complex microstructure. For both the ionic liquid alone and its mixture with rotaxane, a kind of liquid-like stable network is characteristic. This may be due to different electrostatic interactions. The shift angle δ has a constant value of 90° (ideal viscous behavior) for the ionic liquid and 89.5° for the mixture with rotaxane. The frequency sweep test was conducted in the linear viscoelastic region (LVR), as confirmed from the amplitude sweep test; the angular-frequency range was 0.1–100 rad/s (Figure 4).

For both samples the loss modulus (*G*”) dominates the storage modulus (*G*’) over the entire measurement domain. This is an indication of the liquid-like (viscous) character of the systems. The shape of the curves for the mixture (PDMS-Im/Br+PRot) is typical for polymer solutions with a strong dependence on frequency for both moduli. Obviously, no frequency-dependent crossover point of the dynamic moduli appears in the considered frequency range, but it could be supposed at a higher frequency. The addition of rotaxane into the ionic liquid increases the loss modulus by almost three orders of magnitude and the storage modulus by one order.

The next step in the study was to check the influence of temperature on the rheological behavior of the ionic liquid and its mixture with rotaxane. For this purpose an oscillatory temperature test was performed. The temperature-sweep test was carried out in a temperature range between 20 and 80 °C, with a heating rate of 0.5 °C/min, at a constant frequency of 1 Hz and a constant strain amplitude γ = 5%.

**Figure 5:**
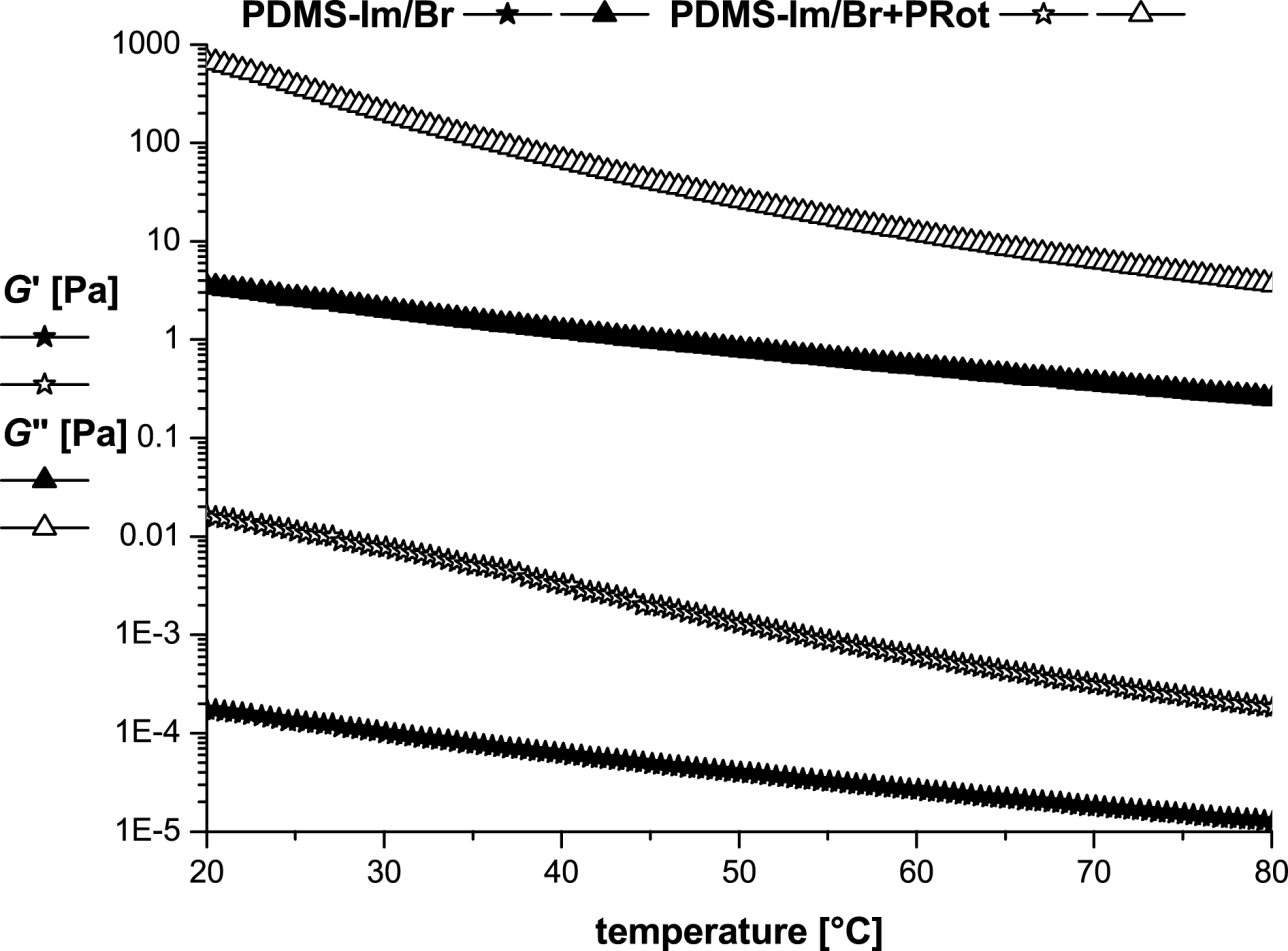
Storage (*G*’) and loss (*G*”) moduli dependence on temperature for PDMS-Im/Br and PDMS-Im/Br+PRot.

As it can be easily seen in Figure 5 the liquid-like stable structure is not disrupted during heating, with *G*’ being almost parallel to *G*” over the entire temperature domain. For both samples the value of the dynamic moduli decreases when the temperature is increased.

**Figure 6:**
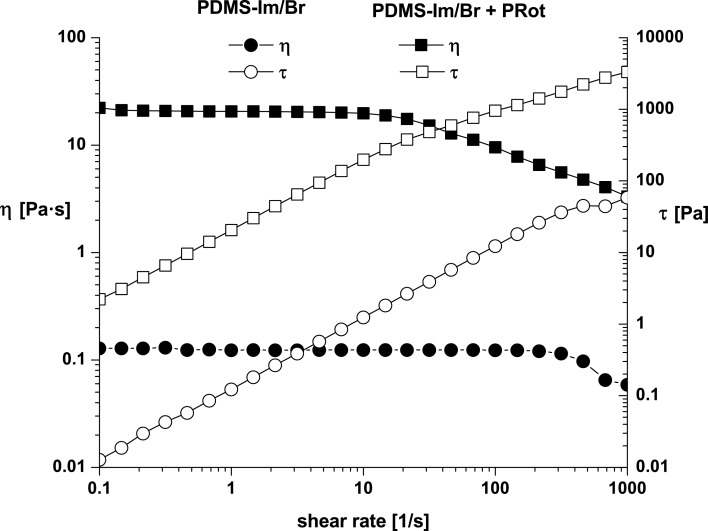
Flow curves for PDMS-Im/Br and PDMS-Im/Br + PRot at 25 °C.

Rotational measurements were also carried out for a better understanding of the rheological behavior of the analyzed samples. The flow curves were recorded both in terms of the shear stress (τ) and viscosity (η) (Figure 6).

The ionic liquid exhibits Newtonian behavior for almost the entire measurement domain, with a viscosity that is independent of the shear rate. A deviation from linearity appears only for high shear rates. An important increase, both in terms of viscosity and shear stress, is noticed when rotaxane is added to the ionic liquid. The first Newtonian domain is obvious as well as the beginning of the shear-thinning behavior. The experimental results were fitted with the Carreau–Yasuda model by using the rheometer software (Rheoplus) [29–30]. The parameters are listed in Table 1.

**Table 1:** Parameters of the Carreau–Yasuda model.

Sample	η_0_ [Pa·s]	η_∞_ [Pa·s]	*a*	*n*	Λ [s]	*R*^2^

PDMS-Im/Br	0.12399	6.99·10^−9^	4.5866	0.2949	0.00288	0.9944
PRot + PDMS-Im/Br	20.654	3.95·10^−7^	2.5312	0.5529	0.05846	0.9994

In this model, η_0_ is the zero-shear viscosity, η_∞_ is infinite-shear viscosity while *a*, *n* and Λ are the regression parameters of the model calculated by using the rheometer software, with Λ being the characteristic relaxation time related to the onset of non-Newtonian behavior or shear-thinning behavior [30]. The experimental data proved to be in very good concordance with the Carreau–Yasuda model.

**Figure 7:**
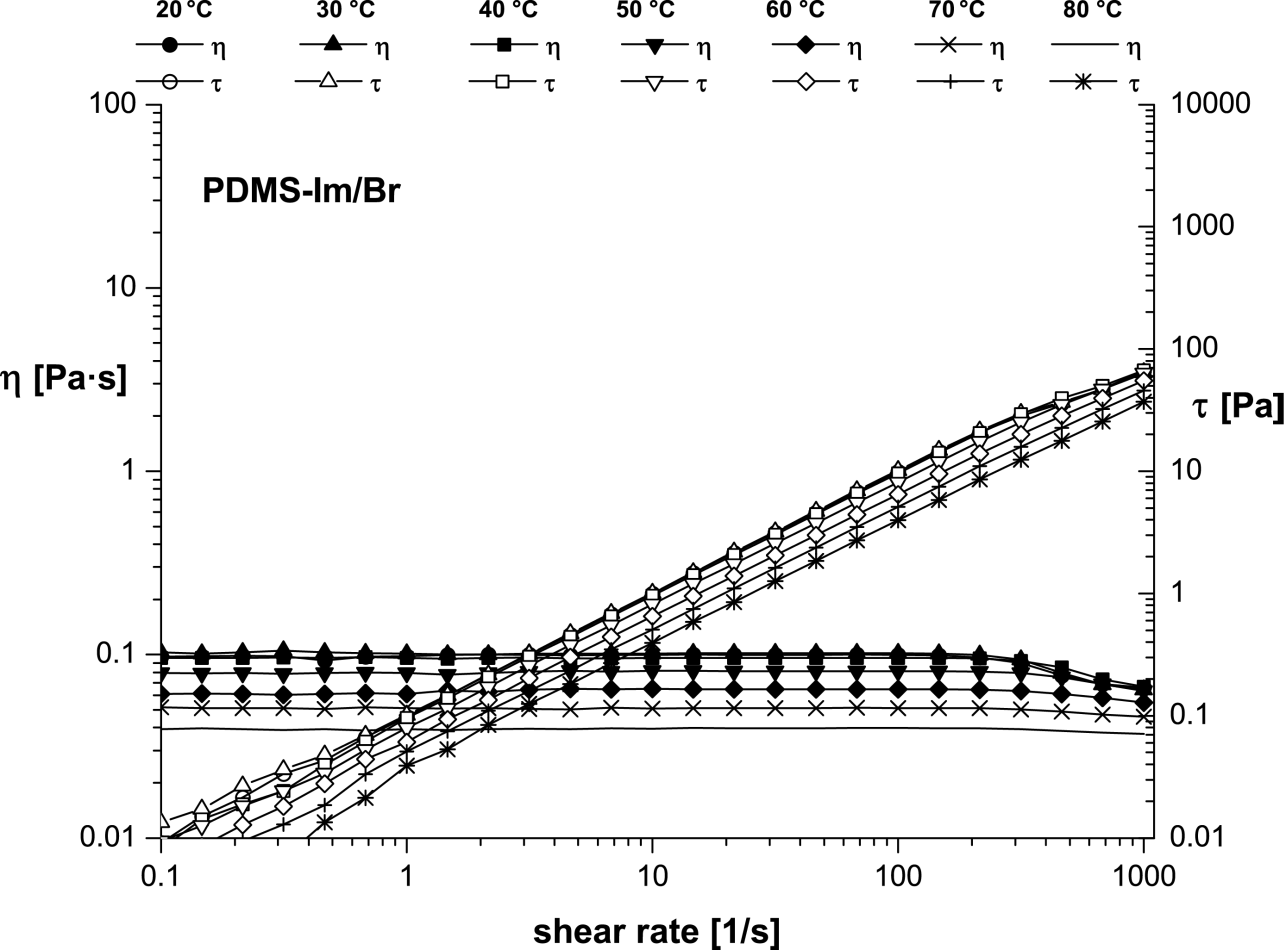
Temperature dependence of flow curves for PDMS-Im/Br ionic liquid.

**Figure 8:**
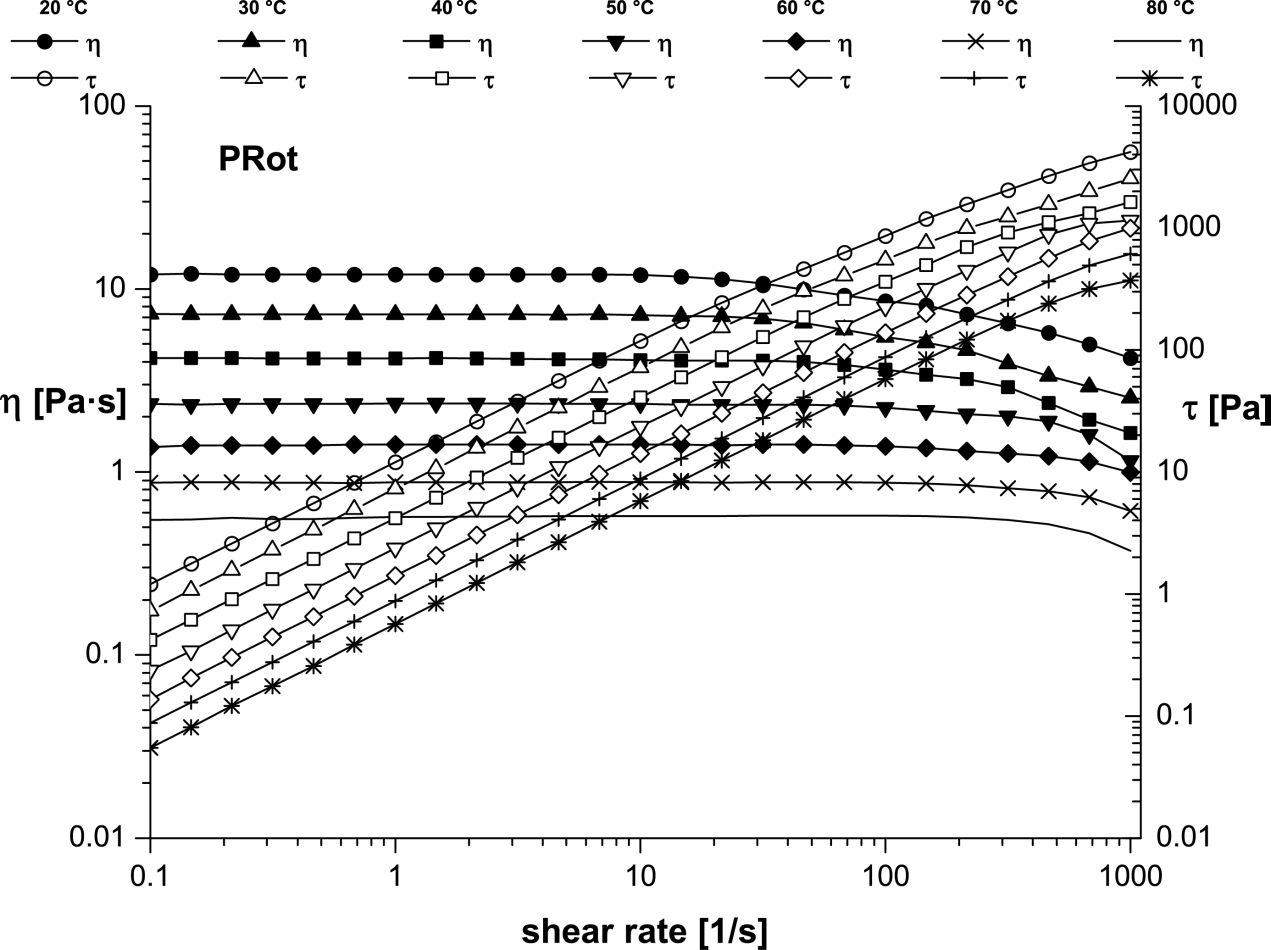
Temperature dependence of flow curves for PDMS-Im/Br+PRot.

Flow curves recorded at seven different temperatures between 20 and 80 °C (Figure 7 and Figure 8) showed a clear increase of the dynamic viscosity for the ionic liquid alone (Figure 7) and even more obviously for the mixture of ionic liquid with rotaxane (Figure 8). This remark concurs with reports in the literature [31–33], namely that the viscosity is strongly temperature-dependent.

**Figure 9:**
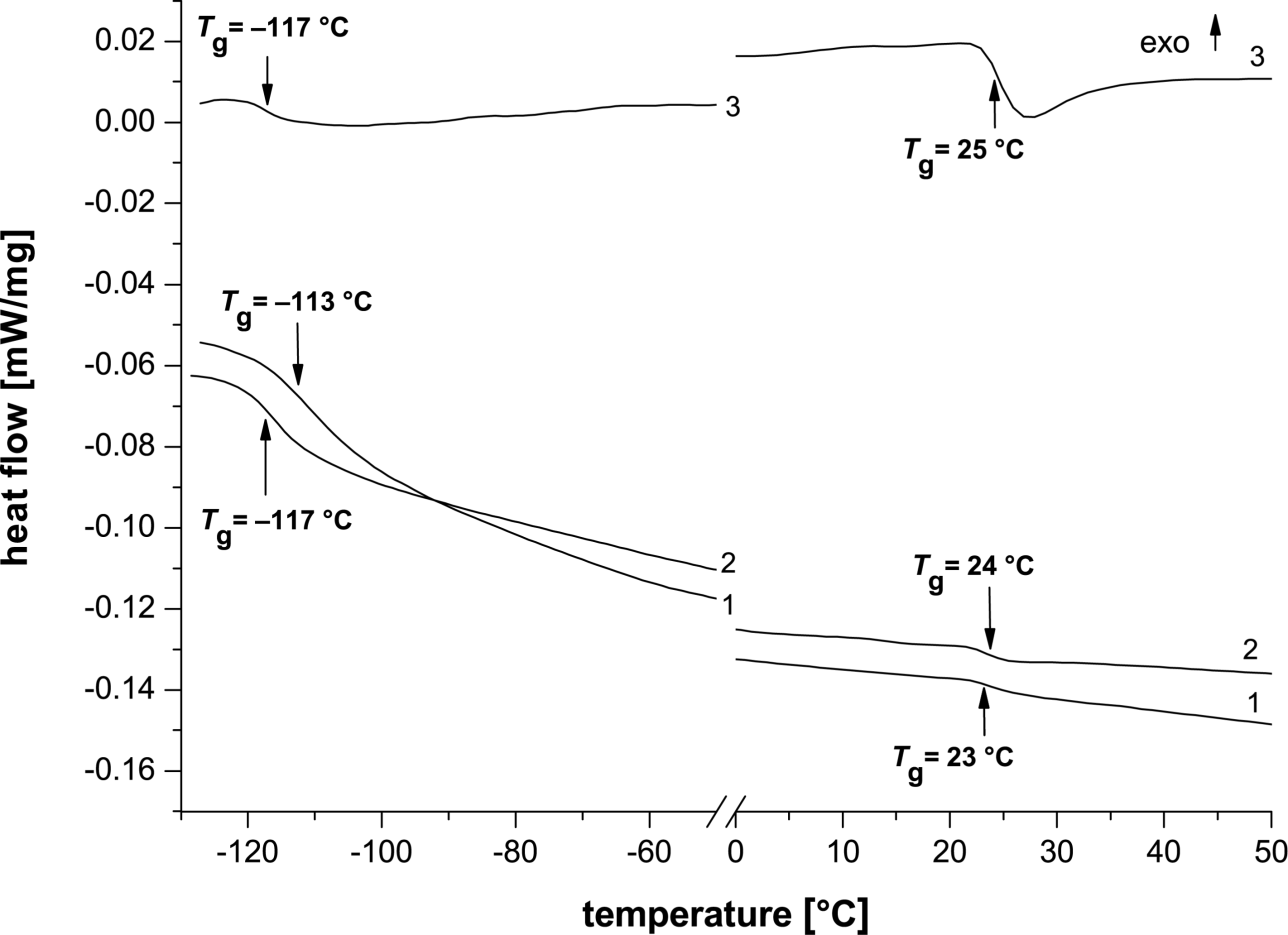
DSC second heating curves of: (1) PDMS-Im/Br ionic liquid, (2) mixture of PDMS-Im/Br with Prot and (3) Prot.

Since the PDMS-Im/Br IL is liquid at room temperature, with *T*_g_ values at −117 and 22 °C, and PRot is in the solid state (*T*_g_: −117 and 25 °C), their mixture presents *T*_g_ values at −112 and 23 °C (Figure 9). In addition, the negative *T*_g_ values are caused by the presence of siloxane chains, while the positive *T*_g_ values are attributed to the imidazolium salt sequences.

## Conclusion

We synthesized, through a multistep procedure (Scheme 1), PDMS-Im/Br ionic liquid with a (CH_3_)_2_SiO/Im(CH_3_)SiO molar ratio of 3/1 and β-CD-polydimethylsiloxane polyrotaxane (PRot) with a β-CD/PDMS chain ratio of 2/1 (PDMS with *M*_n_ = 1250). The obtained ionic liquid proved to be a good solvent for PRot structures (Figure 1).

As expected, the PDMS-Im/Br ionic liquid had a liquid-like non-Newtonian behavior with rheological parameters dependent on frequency and temperature. The addition of rotaxane to the ionic liquid strengthened the non-Newtonian character of the sample and a type of stable liquid-like network was formed due to the contribution of weak ionic interactions. The structure is stable in the 20 to 80 °C domain as proved by the oscillatory and rotational rheological tests.

## Experimental

### Synthesis of ionic liquid

**Synthesis of poly{dimethylsiloxane-co-([5-(1-benzylimidazole-2-yl-oximethyl)pentyl](methyl)siloxane)} (PDMS-Im).** PDMS-lm was synthesized as previously described [22] and the compound was obtained as a yellow viscous product (approximately 78% yield). 2-[(Pent-4-en-1-yl)oxymethyl]-1-benzylimidazole (Allyl-Im, 6.8 g, 0.026 mmol), toluene (10 mL) and Karstedt catalyst (molar ratio: Allyl-Im/Pt(II) 542/1) were placed in a Schlenck reactor. The mixture was stirred at room temperature for 0.5 h. Then, 8.0 g of poly[dimethylsiloxane-co-(H-methyl)siloxane] copolymer (PDMS-H) (molar ratio: SiH(CH_3_)/Allyl-Im 1/1.01) was introduced dropwise at 90 °C. The mixture was stirred at 90 °C for 3 d. The reaction progress was monitored by ^1^H NMR and FTIR spectroscopy by the disappearing of peaks from 4.9 ppm and 2140 cm^−1^, respectively. Finally, the solvent was evaporated and the resulting raw product was purified by column chromatography. ^1^H NMR (400 MHz, CDCl_3_) δ 0.09 (s, 37H, SiC*H*_3_), 0.51 (t, 2H, SiC*H*_2_), 1.25–1.57 (m, 6H, SiCH_2_C*H*_2_C*H*_2_C*H*_2_), 3.39 (t, 2H, CH_2_C*H*_2_O), 4.54 (s, 2H, OC*H*_2_C), 5.23 (s, 2H, NC*H*_2_Ar), 6.88 (s, 1H, =N-C*H*=CHNCH_2_), 7.15 (m, 5H, Ar), 7.26–7.36 (m, 2H, ArCH_2_NC*H=*CH).

**Synthesis of poly{[(1-benzyl-3-*n*-butylimidazole-2-yl-3-ium)methyloxypent-5-yl](methyl)siloxane-co-(dimethylsiloxane)} (PDMS-Im/Br):** The compound was obtained as a brown viscous product (approximately 99% yield). Butyl bromide (5 mL) was added dropwise at 0 °C to a stirred solution of PDMS-Im (26.59 g) in acetonitrile (70 mL). The reaction mixture was stirred for 5 d at 30 °C. The solvent was removed by vacuum distillation [34–35]. FTIR (KBr, cm^−1^): 3064 and 3032 (Ar-H), 2962 and 2858 (C-H), 1676 (C=N), 1456 and 192 (C=C), 1261 (Si-C), 1091–1000 (Si-O-Si), 802 (Si-C); ^1^H NMR (400 MHz, CD_3_OD + D_2_SO_4_) δ 0.01–0.20 (s, SiC*H*_3_), 0.35–0.65 (m, SiC*H*_2_), 0.95–1.05 (s, C-C*H*_3_), 1.25–1.87 (m, SiCH_2_C*H*_2_C*H*_2_C*H*_2_, CH_3_C*H*_2_C*H*_2_CH_2_), 3.35–3.40 (t, CH_2_C*H*_2_OCH_2_), 4.63 (s, OC*H*_2_C), 5.23 (s, NC*H*_2_Ar), 6.98 (s, 1H, CH_2_N-C*H*=CH), 7.15–7.23 (m, 5H, Ar), 7.35–7.55 (m, butyl-NC*H=*CH).

**Synthesis of β-CD-polydimethylsiloxane polyrotaxane (PRot)** was carried out according to a method previously described [17,22] and the compound was obtained as a white powder (approximately 55% yield). PRot was prepared by mixing β-CD in dimethylformamide (DMF, saturated solution) with α,ω-bis(3-glycidoxypropyl)polydimethylsiloxane prepolymer with *M*_n_ = 1250 until the solution became turbid (after approximately 72 h) at 65 °C, followed by the reaction of epoxide functionalities with 4-aminophenyltriphenylmethane (APhTPhM) (saturated solution in isopropyl alcohol) for 8 h at 65 °C. The slurry was then poured into cold water, washed quickly with ethanol, dried, and suspended in diethyl ether overnight to remove the traces of unreacted α,ω-bis(3-glycidoxypropyl)polydimethylsiloxane. After being filtered, the sample was dried at 40 °C for 8 h. The obtained crude PRot contains a significant amount of free β-CD. To remove the noncomplexed β-CD (until the β-CD/siloxane ratio remained unchanged), two successive precipitations in a DMF/water system were made. ^1^H NMR (400 MHz, DMSO-*d*_6_) δ −0.05 (s, C*H*_3_Si chain), 0.04–0.06 (large, CH_3_Si end), 0.44–0.53 (m, CH_2_C*H*_2_Si), 1.46–1.52 (m, C*H*_2_CH_2_Si), 3.32–3.66 (m, C*H*_2_OCH_2_CH(OH)-, C*H*(OH)C*H*_2_NH- and H2–6 from CD), 4.53 (t, OH6 from CD), 4.89 (d, H1 from CD), 5.73–5.77 (two superposed doublets OH2+3 from CD), 6.45–6.47 (d, *ortho* to amino group from APhTPhM), 6.74–6.76 (d, *meta* to amino group from APhTPhM), 7.12–7.29 (m, triphenyl protons from APhTPhM); ^13^C NMR (400 MHz, DMSO-*d*_6_) δ −0.09–0.05 (CH_3_Si chain), 13.30 (CH_2_*C*H_2_Si), 22.60 (*C*H_2_CH_2_Si), 30.46 (tertiary *C* from APhTPhM), 35.58 (CH(OH)*C*H_2_NHPh), 59.70 (C6 from CD), 63.47 (*C*H_2_OCH_2_CH(OH)-), 70.60 (O*C*H_2_CH(OH)CH_2_NH), 71.90, 72.32, 72.64 (C2,3,5 from CD), 72.77 (CH_2_*C*H(OH)CH_2_NH-), 101.40 (C1 from CD), 112.80–162.14 (*C* from APhTPhM).

### Morphological characterization using the Wet-STEM technique

The morphological characterization of samples was performed with a FEI Quanta 200 ESEM. The environmental scanning electron microscope (ESEM) equipped with a Wet-STEM detector enables wet samples to be observed, without potentially damaging them, through the use of partial water vapor pressure in the microscope specimen chamber.

#### Samples preparation

A holey-carbon-coated copper grid was placed on a TEM sample holder and positioned on a Peltier cooling stage. The samples were diluted in THF, and then a small amount of solution was dropped on the grid with a micropipette. The examination of samples was achieved at 1.5 °C, using a gaseous secondary electron detector (GSED) and a STEM detector with two semiannular detectors A and B for bright- or dark-field images. An acceleration voltage of 30 kV was chosen to optimize resolution and sample contrast.

#### Rheological measurements

The rheological measurements were performed on a Physica MCR 501 rheometer (Anton Paar, Austria) with a Peltier device for temperature control, equipped with an electronically commutated synchronous motor, allowing rheological measurements in controlled-stress and controlled-strain modes [29]. To avoid slippage, a parallel plate geometry with serrated plates was used. The upper plate, of stainless steel, was 50 mm in diameter, and a gap of 0.5 mm was fixed. The samples were introduced onto the plate with great care to avoid shear effects in the solutions. A solvent trap was used in all rheological tests to diminish the solvent evaporation. All isothermal measurements were made at 25 °C. Strain sweeps at a fixed frequency of 1 Hz were carried out to establish the limits of the linear viscoelastic region (LVR) both in terms of shear stress and amplitude of deformation. Various rheological parameters were calculated by using the Rheoplus software.

#### Differential scanning calorimetry (DSC)

DSC measurements were conducted on a DSC 200 F3 Maia device (Netzsch, Germany). About 10 mg of each sample was heated in pressed and pierced aluminum crucibles. A heating rate of 10 °C/min was applied. Nitrogen purge gas was used as an inert atmosphere at a flow rate of 50 mL/min. The apparatus was temperature- and sensitivity-calibrated with indium, according to standard procedures.

#### FTIR spectra

FTIR spectra were recorded on a Bruker Vertex 70 FTIR spectrometer from KBr pellets in transmittance mode in the 370–4000 cm^−1^ range, in ambient air at room temperature, with 2 cm^−1^ resolution and accumulation of 32 scans.

#### ^1^H NMR spectra

The ^1^H NMR spectra were recorded on a Bruker Avance DRX 400 spectrometer operating at 400.1 MHz. ^1^H NMR spectroscopy of PDMS-Im was performed in CDCl_3_, and ^1^H NMR spectroscopy of PDMS-Im/Br was performed in fully deuterated methanol (CD_3_OD) with deuterated sulfuric acid (D_2_SO_4_) [35].
